# Facteurs de risque alimentaires du cancer colorectal au Maroc: étude cas témoin

**DOI:** 10.11604/pamj.2020.35.59.18214

**Published:** 2020-02-27

**Authors:** Fatima Ezzahra Imad, Houda Drissi, Nezha Tawfiq, Karima Bendahhou, Abdellatif Benider, Driss Radallah

**Affiliations:** 1 Université Hassan I de Settat, Settat, Maroc; 2 Laboratoire de Biologie et Santé, Unité de Recherche Associée, Centre National pour la Recherche Scientifique et Technique, Urac-34, Faculté des Sciences Ben M’sik Université Hassan II de Casablanca, Casablanca, Maroc; 3 Centre Mohammed VI pour le Traitement des Cancers, Centre Hospitalier et Universitaire, Ibn Rochd, Faculté de Médecine et de Pharmacie, Université Hassan II, Casablanca, Maroc; 4 Registre des Cancers de la Région du Grand Casablanca, Maroc

**Keywords:** Colorectal cancer, case-control study, risk factors, diet, Cancer colorectal, étude cas-témoin, facteurs de risque, régime alimentaire

## Abstract

**Introduction:**

le cancer colorectal par sa fréquence et par sa gravité, est un problème majeur de santé publique. L’alimentation occupe une place primordiale dans la prévention de ce type de cancer. Le but de notre étude est de déterminer, dans notre contexte marocain, les facteurs de risque nutritionnels du cancer colorectal.

**Méthodes:**

il s’agit d’une étude cas témoin incluant les cas du cancer colorectal comparés à des témoins. L’analyse statistique des résultats a été réalisée par le logiciel R.

**Résultats:**

notre étude a inclus 225 patients pris en charge au centre Mohammed VI pour le traitement des cancers et 225 témoins. L’âge moyen de notre population au moment du diagnostic était de 55,49±14,06 ans, dont 119 hommes (52,9%) et 106 femmes (47,1%) avec un sex-ratio de 1,12. Des associations ont été trouvées entre les apports les plus élevés de viandes rouges, de charcuteries, de saucisses et le risque de cancer colorectal avec respectivement ((p = 0,0001); F4 (4-7 fois/semaine) versus F1 (jamais): OR = 4,4 (1,6-11,9); (p = 0,001), OR = 1,7 (0,5-5,7); (p = 0,003), OR = 5,7 (1,2-27,4)). En revanche, la consommation du poisson est associé à une diminution du risque de cancer colorectal (p = 0,0001; OR = 0,3 (0,11-0,7)), alors que la consommation de volailles et des œufs grillés n’est pas associés au cancer colorectal. Nous avons constaté également que l’apport en légumes frais et légumes cuits est faible chez les cas par rapport aux témoins (p = 0,0001). Par ailleurs, l’apport élevé en café noir est associé à une diminution du risque de cancer colorectal (p = 0,0001; F4 versus F1: OR = 0,2 (0,1-0,4)).

**Conclusion:**

notre étude conforte l’idée qu’un changement du régime alimentaire peut prévenir ou empêcher la croissance d’un cancer colorectal. Il est important de promouvoir une alimentation équilibrée, riche en poissons, légumes, fruits et fibres sans excès de viandes rouges et d’éviter les charcuteries et les saucisses.

## Abstract (en)

**Introduction:**

given its frequency and severity, colorectal cancer is a major public health problem. Diet plays a key role in preventing this type of cancer. The purpose of our study was to determine dietary risk factors for colorectal cancer in our Moroccan context.

**Methods:**

we conducted a case-control study including patients with colorectal cancer compared with controls. The statistical analysis of results was carried out using R software.

**Results:**

our study included 225 patients treated for cancer at the Mohammed VI Hospital Center and 225 controls. The average age of our study population at the time of diagnosis was 55.49±14.06 years, including 119 men (52.9%) and 106 women (47.1%) with a sex ratio of 1.12. Associations were found between the highest intakes of red meats, cold meats, sausages and the risk of colorectal cancer (p = 0.0001) with F4 (4-7 times / week) vs F1 (never): OR = 4.4 (1.6-11.9); (p = 0.001), OR = 1.7 (0.5-5.7); (p = 0.003), OR = 5.7 (1.2-27.4)). On the other hand, consumption of fish was associated with a reduced risk of colorectal cancer (p = 0.0001; OR = 0.3 (0.11-0.7)), while consumption of poultry and grilled eggs was not associated with colorectal cancer. We also found that consumption of fresh vegetables and cooked vegetables was low in patients compared to controls (p = 0.0001). Furthermore, a high intake of black coffee was associated with a reduced risk of colorectal cancer (p = 0.0001; F4 vs F1: OR = 0.2 (0.1-0.4)).

**Conclusion:**

our study highlights that dietary changes can prevent or impede the growth of colorectal cancer. It is essential to promote balanced diet, rich in fish, vegetables, fruits and fibers without exceeding recommended levels of red meat and avoiding cold meats and sausages.

## Introduction

Le cancer colorectal (CCR) représente un problème de santé publique par sa fréquence et son mauvais pronostic quand il est diagnostiqué à des stades avancés. Au Maroc, l’incidence du cancer colorectal est à l’heure actuelle en augmentation comme le prouve l’évolution de l’incidence de ce cancer dans la région du grand Casablanca qui est passée de 7,3 cas/100000 habitants en 2004-2007 à 9,6 cas/1000000 habitants en 2008-2012 pour les deux sexes confondus. Il est devenu le premier cancer digestif par son incidence, surclassant le cancer de l’estomac [1]. Ses étiologies sont mal connues, cependant plusieurs facteurs de risque ont été identifiés notamment les facteurs liés à l’environnement et au mode de vie. L’alimentation occupe vraisemblablement une place prépondérante; on estime qu’elle pourrait expliquer jusqu’à 80% des différences d’incidence observées entre les pays [2]. On estime que 70% des cancers colorectaux pourraient être prévenus par une intervention nutritionnelle. Un régime alimentaire très calorique et riche en graisses animales, contenant peu de légumes et de fibres associé à la sédentarité pourrait agir comme facteur promoteur de la carcinogenèse [3]. La situation au Maroc reste mal connue. Peu d’études se sont intéressées à l’évaluation des facteurs de risque alimentaires. L’alimentation est devenue un grand espoir pour la prévention des cancers colorectaux. Il est donc essentiel d’étudier la relation entre l’alimentation et la survenue du CCR dans notre contexte afin de mettre en œuvre une politique de prévention primaire. L’objectif de notre étude est de décrire le comportement alimentaire et la qualité de diète des patients, comparés aux témoins et rechercher ainsi, l’association entre le mode d’alimentation et le risque de survenue du CCR chez la population marocaine.

## Méthodes

**Type d’étude:** il s’agit d’une étude cas témoin menée au sein du Centre Mohammed VI pour le traitement des cancers au Centre Hospitalier IBN ROCHD, l’un des deux plus grands centre pour la prise en charge et le traitement des cancers au Maroc. Un consentement éclairé préalable pour la participation à l’étude est demandé aux participants. Des explications vulgarisées sont données à chacun d’eux à propos des buts de la recherche et de l’intérêt de l’étude.

**Population d’étude:** nous avons inclus dans notre étude de manière consécutive tous les patients atteints de cancers colorectaux confirmés histologiquement et nouvellement diagnostiqués, ayant été pris en charge au centre du 1^er^ janvier 2015 jusqu’au 1^er^ janvier 2017. Le même nombre de témoins indemnes de toute maladie cancéreuse a été inclus parmi les patients admis au centre des consultations de dermatologie et d’ophtalmologie au Centre Hospitalier IBN ROCHD Casablanca. Ce choix s’est fait de façon délibérée pour avoir un échantillon de témoins appartenant à la même classe socioéconomique qui consulte au CHU Ibn Rochd. Les cas ont été appariés aux témoins sur l’âge et le sexe, en utilisant l’âge au diagnostic pour les cas et l’âge à l’entrevue pour les témoins. Le recueil des données a été fait de manière prospective à l’aide d’un questionnaire standardisé comportant plusieurs rubriques de données démographiques, cliniques et surtout alimentaires. Administré en face à face et complété à partir du dossier médical. Ce dernier volet qui regroupe la nature, la diversité et la fréquence des aliments consommés a été conçu et adapté au contexte marocain en se basant sur l’étude European Prospective Investigation into Cancer and Nutrition (Epic) [4]. Au cours de l’enquête, nous avons cherché à connaître la fréquence de consommation de chaque aliment (par mois, par semaine) d’une liste préétablie de l’année qui précède le diagnostic du cancer. Ensuite nous avons stratifié cette fréquence de consommation en quatre catégories: (F1) jamais chez les patients qui ne consomment pas l’aliment demandé, (F2) inférieur à une fois par semaine chez les patients qui consomment l’aliment demandé (1 fois, 2 fois ou 3 fois par mois), (F3) inférieur ou égale à 3 fois par semaine et enfin (F4) supérieur à 3 fois par semaine. La saisie des données a été réalisée par Microsoft Office Excel (2016) et l’analyse des variables par le logiciel R. L’étude d’association en croisant les variables entre les différents groupes a été évaluée par le test de chi-deux. Le test est considérée comme significatif lorsque p<0,05. Les odds ratios (OR) et les intervalles de confiance à 95% (IC) pour les différents aliments chez les cas et les témoins ont été calculés à l’aide d’une analyse de régression logistique.

## Résultats

Durant la période d’étude, il a été inclus un total de 225 patients se présentant pour un cancer colorectal pris en charge au Centre Mohammed VI pour le traitement des cancers, et 225 témoins indemnes de toute maladie cancéreuse. L’âge moyen de la population de notre étude au moment du diagnostic était de 55,49±14,06 ans. Il y avait 119 hommes (52,9%) et 106 femmes (47,1%). Il y avait une prédominance masculine avec un sex-ratio de 1,12. Pour les patients, sur le plan histologique, il ressort de l’analyse des données anatomopathologiques des patients que le CCR touche avec prédilection le rectum dans 50,7% et le côlon dans 49,3% des cas. Le cancer colique a été retrouvé principalement sur le côlon gauche chez 32,9% de nos patients. Le côlon droit vient à la 2^e^ position avec 16,3%. Quant aux types histologiques, l’adénocarcinome lieberkühnien est le type le plus fréquent chez 82% des patients. Les adénocarcinomes bien différenciés sont majoritaires dans notre série, ils représentent 61%. Selon la classification TNM, qui permet d’établir un pronostic de CCR, le stade 2 prédominait avec une fréquence de 39%, suivi de 26% pour le stade 3; néanmoins, 30% des cas présentent des métastases. Dans notre étude, pour le petit déjeuner, seule la moitié des malades le prend régulièrement tous les jours, 57,7% des malades versus 75% des témoins; 36% des patients versus 20% des témoins, quand ils ont le temps et 6,3% des patients versus 5% des témoins ne prennent jamais le petit déjeuner au cours d’une journée de semaine, contre 93,3% versus 98% des témoins qui le prennent régulièrement lors d’une journée libre. Par contre, pour le déjeuner et le diner, il n’y avait pas de différence entre les patients et les témoins comme en témoigne les pourcentages: le déjeuner 99,7% pour les patients versus 100% des témoins, et le diner 88,5% des patients versus 78,7% des témoins. Dans notre étude, la consommation des viandes rouges est significativement élevée, 23,1% des patients interrogés versus 9,8% des témoins en mangent de façon quotidienne, 60,9% des patients versus 56,9% des témoins de façon hebdomadaire et 12,4% des patients versus 26,7% des témoins de façon mensuelle; la différence était significative (p = 0,0001). En outre, 12% des patients versus 4,4% des témoins ont déclaré qu’ils mangent la charcuterie de façon hebdomadaire (p = 0,001). Le risque de développer un CCR est estimé à 4,4 (1,6-11,9) chez ceux qui consomment la viande rouge quotidiennement par rapport à ceux qui n’en consomment pas. Concernant la consommation de la viande blanche, 36% des patients versus 41,8% des témoins enquêtées en mangent hebdomadairement sans que la différence soit significative. Le risque de cancer colorectal n’était pas corrélé avec la viande blanche.

Pour le poisson, 6,7% des patients versus 13,3% des témoins en consomment quotidiennement, 24,9% des patients contre 43,1% des témoins hebdomadairement et 59,6% des patients versus 38,2% des témoins mensuellement avec une différence significative (p = 0,0001). L’apport élevé du poisson était associé à une diminution du risque de CCR (F4 versus F1: OR = 0,30; IC: 0,11-0,7). Quant aux œufs grillés, ils sont consommés quotidiennement par 14,7% des patientes versus 11,6% des témoins. Le risque de cancer colorectal n’était pas corrélé avec les œufs grillés (Tableau 1). Par ailleurs, la consommation journalière des légumes crus et cuits est moins importante chez les patients que chez les témoins avec respectivement 10,2% des patients versus 23,6% des témoins et 19,6% des patients versus 29,8% des témoins et pour la consommation hebdomadaire 20% des cas contre 42,2% des témoins pour les légumes crus et 48% des cas versus 46,7% des témoins pour les légumes cuits. Chez les sujets qui consomment quotidiennement les légumes; le risque estimé de ce cancer était faible pour les légumes cuits (p = 0,01; OR = 0,32; IC: 0,16-0,6) et pour les légumes crus (p = 0,0001; OR=0,12; IC: 0,06-0,2). Parallèlement, nos cas de CCR consomment de façon globale plus fréquemment les pommes de terre cuites que les témoins avec p = 0,001. L’estimation du risque de CCR pour les patients qui consomment les frites et pomme de terre étaient élevée avec respectivement F1 versus F4: OR = 2,3; OR = 12,3. Nos résultats ont révélé que la consommation journalière des fruits par les patients est faible, par contre celle des témoins est importante avec (p = 0,0001). Avec des apports croissants de fruits frais, le risque de cancer colorectal a montré une baisse de 58% (OR= 0,42).

**Tableau 1:** Répartition des cas et témoins selon la consommation des viandes et dérivés

	Cas (%)	Témoins (%)	p-value	OR	IC95%
**Viandes rouges**					
F1: Jamais	8(3,6)	15(6,7)	0,0001		
F2: 1-3 fois / mois	28(12,4)	60(26,7)		0,8	0,3-2,3
F3: 1-3 fois / semaine	137(60,9)	128(56,9)		2,1	0,8-4,8
F4: 4-7 fois / semaine	52(23,1)	22(9,8)		4,4	1,6-11,9
**Charcuterie**					
F1: Jamais	141(62,7)	181(80,4)	0,001		
F2: 1-3 fois / mois	50(22,2)	29(12,9)		2,2	1,3-3,6
F3: 1-3 fois / semaine	27(12)	10(4,4)		3,4	1,6-7,3
F4: 4-7 fois / semaine	7(3,1)	5(2,2)		1,7	0.5-5,7
**Saucisses**					
F1: Jamais	105(46,7)	150(66,7)	0,003		
F2: 1-3 fois / mois	82(36,4)	62(27,6)		1,9	1,2-2,8
F3: 1-3 fois / semaine	30(13,3)	11(4,9)		3,9	1,8-8,1
F4: 4-7 fois / semaine	8(3,6)	2(0,9)		5,7	1,2-27,4
**Foie**					
F1: Jamais	152(67,6)	116(51,6)	0,007		
F2: 1-3 fois / mois	52(23,1)	75(33,3)		0,4	0,13-1,2
F3: 1-3 fois / semaine	16(7,1)	25(11,1)		0,81	0,2-2,5
F4: 4-7 fois / semaine	5(2,2)	9(4)		0,86	0,2-3,06
**Abats**					
F1: Jamais	131(58,2)	169(75,1)	----		
F2: 1-3 fois / mois	79(35,1)	45(20)		0.7	0.04-12.5
F3: 1-3 fois / semaine	14(6,2)	10(4,4)		1.75	0.1-28.7
F4: 4-7 fois / semaine	1(0,4)	1(0,4)		1.4	0.07-25.1
**Viandes blanches**					
F1: Jamais	7(3,1)	14(6,2)			
F2: 1-3 fois / mois	124(55,1)	103(45,8)	0,15	2,4	0,9-6,1
F3: 1-3 fois / semaine	81(36)	94(41,8)		1,7	0,6-4,4
F4: 4-7 fois / semaine	13(5,8)	14(6,2)		1,8	0,5-6,04
**Poissons**					
F1: Jamais	20(8,9)	12(5,3)			
F2: 1-3 fois / mois	134(59,6)	86(38,2)	0,0001	0,9	0,4-2,01
F3: 1-3 fois / semaine	56(24,9)	97(43,1)		0,35	0,15-0,7
F4: 4-7 fois / semaine	15(6,7)	30(13,3)		0,30	0,11-0,7
**Œufs durs**					
F1: Jamais	132(58,7)	110(48,9)			
F2: 1-3 fois / mois	60(26,7)	65(28,9)	0,07	0,7	0,5-1,2
F3: 1-3 fois / semaine	25(11,1)	43(19,1)		0,4	0,2-0,8
F4: 4-7 fois / semaine	8(3,6)	7 (3,1%)		0,9	0,3-2,7
**Œufs grillés**					
F1: Jamais	75(33,3)	61(27,1)			
F2: 1-3 fois / mois	60(26,7)	87(38,7)	0,06	0,5	0,3-0,8
F3: 1-3 fois / semaine	57(25,3)	51(22,7)		0,9	0,5-1,5
F4: 4-7 fois / semaine	33(14,7)	26(11,6)		1,01	0,5-1,8

Cependant, les patients consomment moins les fruits secs et oléagineux que les témoins avec une différence significative, respectivement p = 0,001; p = 0,004. La consommation de fruits secs et de fruits oléagineux est associé à une diminution du risque de ce cancer avec respectivement (OR = 0,2; IC= 0,07-0,48); (OR = 0,2; IC = 0,09-0,6) (Tableau 2). En outre, nous avons noté que le thé est la boisson le plus consommée par les patients que les témoins avec p = 0,009. Les apports de café au lait, infusion et soda ont été similaires chez les patients et les témoins, tandis que 17,3% des témoins versus 7,6% des patients déclaraient siroter le café journalièrement avec p = 0,001. Ainsi, le risque de cancer colorectal est ainsi associé avec la consommation de café (F4 versus F1: OR = 0,2) (Tableau 3). Concernant l’apport en produits laitiers, les cas et les témoins boivent le lait hebdomadairement presque de la même fréquence, 19,1% des cas versus 18,7% des témoins (p = 0,07). Les patients consomment moins de fromage fondu, de fromage à pâte pressée type fromage rouge) et de fromage frais traditionnel *«Jben»* par rapport aux témoins, avec respectivement p = 0,3; p = 0,00; p = 0,001 (Tableau 4). Pour le riz, les patients consomment moins de riz par rapport aux témoins avec une différence significative (p = 0,001), ce qui est associé à une diminution de risque de CCR estimé à 0,08; IC: 0,02-0,2 (Tableau 5). Les patients ont signalé des apports plus élevés de gâteaux et de mesemen que les témoins, avec respectivement p = 0,04; p = 0,08. Des associations ont été trouvées entre les apports les plus élevés de gâteaux, de pâtes et le risque de cancer colorectal (F4 versus F: OR = 1,33; OR= 1,28) (Tableau 5 et Tableau 6). Par ailleurs, des associations ont été trouvées entre les apports les plus élevés des pâtisseries à la crème et le risque de cancer colorectal (p= 0,001; F4 versus F: OR = 3,9; IC: 1,2-12,5) (Tableau 6). Concernant les habitudes toxiques, la consommation d’alcool était retrouvée dans 11,55% des cas, contre 1,33% des témoins. Ces résultats indiquent une association significative entre l’alcool et la survenue de cancer colorectal (P=0,0001).

**Tableau 2:** Répartition des cas et témoins selon la consommation des fruits et légumes et dérivés

	Cas (%)	Témoins (%)	p-value	OR	IC95%
**Légumes crus**					
F1: Jamais	70(31,1)	20(8,9)	0,0001		
F2: 1-3 fois / mois	87 (38,7)	57(25,3)		0,43	0,2-0,7
F3:1-3 fois / semaine	45(20)	95(42,2)		0,13	0,07-0,2
F4: 4-7 fois / semaine	23(10,2)	53(23,6)		0,12	0,06-0,2
**Légumes cuits**					
F1: Jamais	36(16)	18(8)	0,01		
F2: 1-3 fois / mois	37(16,4)	35(15,6)		0,53	0,2-1,09
F3: 1-3 fois / semaine	108(48)	105(46,7)		0,51	0,2-0,9
F4: 4-7 fois / semaine	44(19,6)	67(29,8)		0,32	0,16-0,6
**Légumes secs**					
F1: Jamais	55(24,4)	55(24,4)	0,8		
F2: 1-3 fois / mois	146(64,9)	139(61,8)		1,07	0,6-1,6
F3: 1-3 fois / semaine	19(8,4)	22(9,8)		0,8	0,4-1,8
F4: 4-7 fois / semaine	6(2,7)	9(4)		0,7	0,2-2,03
**Pomme de terre**					
F1: Jamais	15(6,7)	50(22,2)	0,001		
F2: 1-3 fois / mois	63(28)	97(43,1)		2,1	1,1-4,1
F3: 1-3 fois / semaine	121(53,8)	71(31,6)		5,6	2,9-10,8
F4: 4-7 fois / semaine	26(11,6)	7(3,1)		12,3	4,4-34,1
**Frites**					
F1: Jamais	74(32,9)	70(31,1)	----		
F2: 1-3 fois / mois	120(53,3)	145(64,4)		0,7	0,5-1,1
F3: 1-3 fois / semaine	26(11,6)	8(3,6)		3,07	1,3-7,2
F4: 4-7 fois / semaine	5(2,2)	2(0,9)		2,3	0,4-12,5
**Fruits**					
F1: Jamais	7(3,1)	6 (2,7)			
F2: 1-3 fois / mois	72(32)	38 (16,9)	0,0001	1,6	0,5-5,1
F3: 1-3 fois / semaine	109(48,4)	105(46,7)		0,8	0,2-2,7
F4: 4-7 fois / semaine	37(16,4)	76(33,8)		0,42	0,1-1,3
**Fruits oléagineux**					
F1: Jamais	191(84,9)	162(72)			
F2: 1-3 fois / mois	24(10,7)	35(15,6)	0,004	0,5	0,3-1,01
F3: 1-3 fois / semaine	5(2,2)	17(7,6)		0,2	0,09-0,6
F4: 4-7 fois / semaine	5(2,2)	11(4,9)		0,3	0,1-1,1
**Fruits secs**					
F1: Jamais	127(56,4)	106(47,1)			
F2: 1-3 fois / mois	64(28,4)	56(24,9)	0,001	0,94	0,6-1,4
F3: 1-3 fois / semaine	28(12,4)	37(16,4)		0,6	0,3-1,1
F4: 4-7 fois / semaine	6(2,7)	26(11,6)		0,2	0,07-0,48

**Tableau 3:** Répartition des cas et témoins selon la consommation des boissons

	Cas (%)	Témoins (%)	p-value	OR	IC95%
**Thé**					
**F1**: Jamais	4(1,8)	14(6,2)	0,009		
**F2**: 1-3 fois / mois	15(6,7)	19(8,4)		2,7	0,7-10,1
**F3**: 1-3 fois / semaine	45(20,1)	61(27,1)		2,5	0,7-8,3
**F4**: 4-7 fois / semaine	161(71,4)	131(58,2)		4,3	1,3-13,3
**Café au lait**					
**F1**: Jamais	88(39,3)	84(37,3)	0,4		
**F2**: 1-3 fois / mois	57(25)	45(20)		1,2	0,7-1,9
**F3**: 1-3 fois / semaine	30(13,4)	33(4,7)		0,8	0,4-1,5
**F4**: 4-7 fois / semaine	50(22,3)	63(28)		0,7	0,4-1,2
**Café noir**					
**F1**: Jamais	134(59,4)	79(35,1)	0,001		
**F2**: 1-3 fois / mois	31(13,8)	48(21,3)		0,3	0,2-0,6
**F3**: 1-3 fois / semaine	43(19,2)	59(26,2)		0,4	0,2-0,6
**F4**: 4-7 fois / semaine	17(7,6)	39(17,3)		0,2	0,1-0,4
**Jus de fruits**					
**F1**: Jamais	121(54)	116(51,6)	0,6		
**F2**: 1-3 fois / mois	48(21,4)	41(18,2)		1,1	0,6-1,8
**F3**: 1-3 fois / semaine	38(16,9)	48(21,3)		0,7	0,4-1,2
**F4**: 4-7 fois / semaine	18(8)	20 (8,9)		0,8	0,4-1,7
**Soda**					
**F1**: Jamais	74(33)	92(40,9)	0,3		
**F2**: 1-3 fois / mois	77(34,4)	72(32)		1,3	0,8-2,09
**F3**: 1-3 fois / semaine	40(17,9)	39(17,3)		1,2	0,7-2,1
**F4**: 4-7 fois / semaine	33(14,7)	22(9,8)		1,8	1-3,4
**Infusion**					
**F1**: Jamais	164(72,8)	154(68,4)			
**F2**: 1-3 fois / mois	26(11,6)	34(15,1)	0,4	0,7	0,4-1,2
**F3**: 1-3 fois / semaine	24(10,7)	30(13,3)		0,7	0,4-1,3
**F4**: 4-7 fois / semaine	11(4,9)	7(3,1)		1,4	0,5-3,9

**Tableau 4:** Répartition des cas et témoins selon la consommation des produits laitiers et dérivés

	Cas (%)	Témoins (%)	p-value	OR	IC95%
**Lait**					
**F1**: Jamais	84(37,3)	95(42,5)	**0,07**		
**F2**: 1-3 fois / mois	77(34,2)	74(24)		1,6	1,02-2,5
**F3**: 1-3 fois / semaine	42(18,7)	43(19,1)		1,1	0,6-0,8
**F4**: 4-7 fois / semaine	22(9,8)	33(14,7)		0,7	0,4-1,3
**Lben**					
**F1**: Jamais	124(55,1)	111(49,3)	**0,5**		
**F2**: 1-3 fois / mois	42(18,7)	52(23,1)		0,7	0,4-1,1
**F3**: 1-3 fois / semaine	52(23,1)	56(24,9)		0,8	0,5-1,3
**F4**: 4-7 fois / semaine	7(3,1)	6(2,7)		1,04	0,3-3,2
**Yaourt**					
**F1**: Jamais	74(32,9)	63(28)	**0,3**		
**F2**: 1-3 fois / mois	65(28,9)	57(25,3)		0,9	0,5-1,5
**F3**: 1-3 fois / semaine	59(26,2)	69(58,7)		0,7	0,4-1,1
**F4**: 4-7 fois / semaine	27(12)	36(16)		0,6	0,3-1,1
**Fromages fondus**					
**F1**: Jamais	67(29,8)	53(23,6)	**0,3**		
**F2**: 1-3 fois / mois	65(28,9)	60(26,7)		0,8	0,5-1,4
**F3**: 1-3 fois / semaine	48(21,3)	58(25,8)		0,6	0,3-1,1
**F4**: 4-7 fois / semaine	45(20)	54(24)		0,6	0,3-1,1
**Fromages rouges**					
**F1**: Jamais	122(54,2)	96(42,7)	**0,001**		
**F2**: 1-3 fois / mois	70(31,1)	57(25,3)		0,9	0,6-1,5
**F3**: 1-3 fois / semaine	22(9,8)	48(21,3)		0,3	0,2-0,6
**F4**: 4-7 fois / semaine	11(4,9)	24(10,7)		0,3	0,1-0,7
**Crème**					
**F1**: Jamais	183(81,8)	161(71,6)	**0,08**		
**F2**: 1-3 fois / mois	25(11,1)	40(17,8)		0,5	0,3-0,9
**F3**: 1-3 fois / semaine	11(4,9)	18(8)		0,5	0,2-1,1
**F4**: 4-7 fois / semaine	5(2,2)	6(2,7)		0,7	0,2-2,4
**Jben**					
**F1**: Jamais	164(72,9)	173(76,9)			
**F2**: 1-3 fois / mois	39(21,8)	24(10,7)	**0,001**	2,1	1,2-3,6
**F3**: 1-3 fois / semaine	19(4)	23(10,2)		0,4	0,1-0,9
**F4**: 4-7 fois / semaine	3(1,3)	5(2,2)		0,6	0,1-2,6

**Tableau 5:** Répartition des cas et témoins selon la consommation des pâtes et céréales raffinés

Variables	Cas (%)	Témoins (%)	p-value	OR	IC95%
**Mesemen**					
**F1**: Jamais	50(22,2)	40(17,8)	0,08		
**F2**: 1-3 fois / mois	65(28,9)	91(40,4)		0,5	0,3-0,9
**F3**: 1-3 fois / semaine	78(34,7)	68(30,2)		0,9	0,5-1,5
**F4**: 4-7 fois / semaine	32(14,2)	26(11,6)		0,9	0,5-1,9
**Viennoiseries**					
**F1**: Jamais	111(49,3)	95(42,2)	0,2		
**F2**: 1-3 fois / mois	67(29,8)	67(29,8)		0,8	0,5-1,3
**F3**: 1-3 fois / semaine	37(16,4)	47(20,9)		0,6	0,4-1,1
**F4**: 4-7 fois / semaine	10(4,4)	16(7,1)		0,5	0,2-1,2
**Pâtes**					
**F1**: Jamais	55(24,4)	55(24,4)	0,8		
**F2**: 1-3 fois / mois	145(64,9)	139(61,8)		1,04	0,6-1,6
**F3**: 1-3 fois / semaine	19(8,4)	22(9,8)		0,8	0,4-1,7
**F4**: 4-7 fois / semaine	6(2,7)	9(4)		0,6	0,2-2
**Riz**					
**F1**: Jamais	50(22,2)	15(6,7)	0,001		
**F2**: 1-3 fois / mois	96(42,7)	121(53,8)		0,2	0,1-0,4
**F3**: 1-3 fois / semaine	72(32)	63(28)		0,3	0,1-0,6
**F4**: 4-7 fois / semaine	7(3,1)	26(11,6)		0,08	0,02-0,2
**Couscous**					
**F1**: Jamais	19(8,5)	21(9,3)	**------**		
**F2**: 1-3 fois / mois	112(49,8)	129(57,3)		----	--------
**F3**: 1-3 fois / semaine	93(41,3)	75(33,3)		----	--------
**F4**: 4-7 fois / semaine	1(0,4)	0(0)		----	--------

**Tableau 6:** Répartition des cas et témoins selon la consommation des gâteaux et sucreries

Chocolats	Cas (%)	Témoins (%)	p-value	OR	IC95%
F1: Jamais	16(7,1)	10(4,4)	0,6		
F2: 1-3 fois / mois	109(48,4)	97(43,1)		0,01	0,01-0,03
F3: 1-3 fois / semaine	25(11,1)	45(20)		0,05	0,02-0,1
F4: 4-7 fois / semaine	75(33,3)	73(32,4)		0,09	0,04-0,2
**Biscuits secs**					
F1: Jamais	161(71,6)	172(76,8)	0,6		
F2: 1-3 fois / mois	31(13,8)	25(11,1)		1,3	0,7-2,3
F3: 1-3 fois / semaine	24(10,7)	22(9,8)		1,1	0,6-2,1
F4: 4-7 fois / semaine	9(4)	6(2,7)		1,6	0,5-4,6
**Gâteaux maison**					
F1: Jamais	75(33,3)	73(32,4)	0,04		
F2: 1-3 fois / mois	109(48,4)	97(43,1)		1,09	0,7-1,6
F3: 1-3 fois / semaine	25(11,1)	45(20)		0,5	0,3-0,9
F4: 4-7 fois / semaine	16(7,1)	10(4,4)		1,5	0,6-3,6
**Gâteaux pâtisseries**					
F1: Jamais	139(61,8)	120(53,3)	0,2		
F2: 1-3 fois / mois	49(21,8)	64(28,4)		0,6	0,4-1,03
F3: 1-3 fois / semaine	21(9,3)	21(9,3)		0,8	0,4-1,6
F4: 4-7 fois / semaine	16(7,1)	20(8,9)		0,6	0,3-1,3
**Pâtisseries à la crème**					
F1: Jamais	159(70,7)	211(93,8)	0,001		
F2: 1-3 fois / mois	38(16,9)	6(2,7)		8,4	3,4-20,3
F3: 1-3 fois / semaine	16(7,1)	4(1,8)		5,3	1,7-16,2
F4: 4-7 fois / semaine	12(5,3)	4(1,8)		3,9	1,2-12,5
**Tartes aux fruits**					
F1: Jamais	152(67,6)	147(65,3)	0,9		
F2: 1-3 fois / mois	46(20,4)	52(23,1)		0,8	0,5-1,3
F3: 1-3 fois / semaine	19(8,4)	19(8,4)		0,9	0,4-1,9
F4: 4-7 fois / semaine	8(3,6)	7(3,1)		1,1	0,3-3,1

## Discussion

Cette étude réalisée au Maroc a permis l’exploration de facteurs alimentaires relatifs au cancer colorectal. Plusieurs études confirment que la désorganisation du rythme des repas a été associée à une augmentation de risque de cancer colorectal [5–7]. Cette association a été retrouvée également dans notre étude. Ces effets pourraient s’expliquer par un flux d’acides biliaires primaires à chaque initiation du processus de digestion ou par l’intervention de l’insuline produite à chaque pic glycémique [7]. D’après nos résultats, la viande rouge est un facteur de risque pour le CCR (p = 0.0001). Notre étude concorde avec plusieurs études dont celle de EPIC «European Prospective Investigation into and Nutrition» qui a prouvé une forte corrélation entre la consommation de viandes rouges et les charcuteries et le risque du CCR. Ce risque est plus élevé d’un tiers chez les sujets qui en consomment régulièrement par rapport à ceux qui en consomment moins [8]. Ainsi, le Centre International de Recherche sur le Cancer (CIRC) a évalué les résultats de 800 études démontrant cette forte association de la consommation de ces deux aliments et CCR [9, 10]. Cet effet nocif est dû à la forte teneur en graisses saturées et en fer qui oxyde les lipides de notre régime alimentaire, formant des composés toxiques qui attaquent les cellules épithéliales du côlon et favorisent la carcinogénèse [11]. L’effet qui a reçu le plus d’attention jusqu’à présent est la présence d’hydrocarbures aromatiques polycycliques, reconnues comme cancérigènes; leur rôle dépend grandement des méthodes de cuisson des viandes à activer ces amines hétérocycliques en composés mutagènes (N-acétylation), ainsi que l’effet renforçateur du (nitrosyl) hème sur la formation de composés N-nitroso cancérigènes et la peroxydation lipidique [12–14].

Nos résultats met en évidence un effet protecteur de la consommation du foie sur le risque de ce cancer, cet aliment de moins en moins présents dans nos assiettes est riche en protéines, en minéraux essentiels tels que le sélenium, le phosphore, le potassium, le magnésium, le calcium et le fer et en vitamines A, D. Le foie, est assez pauvre en lipides. Il contient moins de 10% de matières grasses. Le foie peut donc être consommé en remplacement d’une viande rouge. Il convient cependant d’être attentif à l’impact du mode de préparation, en particulier la salaison et la cuisson, afin de limiter le risque de développement du cancer colorectal. Par ailleurs, nos résultats montrent que la consommation de la viande blanche est presque similaire entre patients et témoins. Cette donnée est comparable avec une méta-analyse (16 cas-témoins et 5 cohortes) qui a montré une association nulle entre le CCR et la consommation de la viande blanche [15]. D’après nos résultats, on constate un effet protecteur des poissons contre le CCR. Ceci est prouvé par l’étude de Nayak *et al*. et celle de Norat *et al*. qui ont montré que ces aliments diminuent le risque du CCR [8, 16]. Egalement, une étude menée sur plus de 1600 patients, évoque un impact positif potentiel de la consommation des poissons sur CCR [17]. La consommation régulière de poisson permet d’apporter à l’organisme des acides gras oméga-3 anti-inflammatoires et des anti-cancéreux, ce qui potentialise l’effet protecteur des végétaux. Sans compter que le poisson est une des rares sources alimentaires de vitamine D, une vitamine connue pour réduire le risque de cancer colorectal [18]. Conformément à d’autres études, comme l’étude EPIC [19], nos résultats soulignent que les fruits et les légumes confèrent une protection contre le CCR. Ce rôle protecteur vis-à-vis du CCR est confirmé par d’autres études, une égyptienne [20] et une étude cas-témoins coréenne [21], avec des OR respectivement de 0,11 et 0,33. Dans une étude belge [22], le rôle protecteur des légumes verts crus est un peu plus net que celui des légumes verts cuits, ce qui s’accorde avec nos résultats.

Les fruits et légumes constituent l’une des principales sources en fibres, vitamines, minéraux et autres composants biologiquement actifs [23]. Leur effet protecteur pourrait s’expliquer par l’augmentation de la masse et de la viscosité du contenu colique, entrainant d’une part une diminution de la concentration des substances cancérogènes ou promotrices présentes dans la lumière colique et de leur diffusion vers la paroi, et d’autre part une réduction du temps de transit, entrainant une réduction du temps de production de métabolites cancérogènes ou promoteurs par les bactéries, ainsi que leur temps de contact avec la muqueuse [24, 25]. Les fruits secs et oléagineux sont un excellent moyen de satisfaire nos besoins nutritionnels, particulièrement en deux micronutriments tels que les omégas 3 et le magnésium. Nos résultats sont cohérents avec ceux de la littérature; consommer régulièrement des fruits à coque, comme les amandes ou les noix, baisse de 57% le risque de ce cancer et réduit de plus de 40% le risque de résurgence du cancer du côlon. Grâce à leur teneur en antioxydants mais surtout en fibres, les noix et les amandes participent à la prévention du cancer colorectal [26, 27]. Une attention particulière est portée à la consommation du lait, nos résultats (p=0,4) sont en désaccord avec plusieurs études [28]. Cho *et al.* ainsi que Dehlavi *et al.* concluent à un effet protecteur du calcium en ralentissant la croissance des cellules cancéreuses et la croissance des vaisseaux sanguins dans les tumeurs colorectales [29, 30]. Selon l’étude européenne EPIC, des niveaux sanguins élevés de vitamine D sont associées à une réduction de 40% du risque de cancer colorectal [31]. Des études observationnelles ont montré qu’il existe un lien entre les concentrations élevées de 25(OH)D et la réduction de 50% du risque de CCR [31, 32], en freinant la prolifération des cellules tumorales colorectales [33]. Conformément à nos résultats, plusieurs travaux scientifiques ont mis en évidence une association inverse entre la consommation des produits laitiers fermentés en particulier le yaourt et le risque du CCR [34].

Le thé vert est connu être un allié contre le cancer colorectal. En effet, il a été montré que la consommation d’un verre de thé par semaine réduit le risque de CCR avec un OR de 0,37 [28, 35]. Le thé contient des polyphénols et d’autres substances anti-oxydantes telles les catéchines, surtout l’épigallocatéchine gallate, qui pourraient inhiber le développement d’un cancer et bloquer les lésions de l’ADN cellulaire. Cependant, nos résultats n’ont pas mis en faveur cette association [35]. Parallèlement, le café est considéré comme une arme naturelle contre le cancer colorectal. Nos résultats évoquent cet impact positif du café sur le CCR et sont soutenus par la littérature; Schmit a constaté que la consommation de café a entraîné une diminution de plus de 25% du risque de développer un cancer colorectal avec un OR = 0,76 [36]. Une autre étude fait état d’un risque réduit d’environ 17% du cancer colorectal lié à la consommation de café et jusqu’à 30% pour les plus grands consommateurs [37]. Cette protection apportée par le café serait liée soit aux propriétés anticarcinogènes des diterpènes et antioxydants du café soit à la propriété du café d’induire l’excrétion d’acides biliaires et de stérols neutres dans le colon, de stimuler la motilité colique et d’inhiber la croissance des cellules cancéreuses du colon [38].

Dans notre étude, nous avons noté une association positive entre la consommation des produits issus de céréales raffinés (pates, couscous, mesemen) et le risque de CCR. Ces résultats sont en accord avec plusieurs études sur la consommation de grains entiers, les céréales raffinées et les pates, notamment celles réalisées aux Etats Unis [39] et en Jordanie [40]. Nos résultats rapportent également que la consommation de sucreries et gâteaux est retrouvée dans des proportions élevées chez les cas que chez les témoins. Dans 14 études consacrées à l’effet du sucre, sept retrouvent une association positive, dont une étude met en évidence l’association des pâtisseries et CCR. Les mécanismes pour expliquer cette association incriminent en particulier l’hyperinsulinisme [41, 42]. Aussi, l’augmentation de la consommation en sucres raffinés et en farines blanches est de nature à favoriser les facteurs d’inflammation [43]. L’alcool est un agent cancérigène connu qui peut être associé au cancer colorectal, en baissant le niveau d’acide folique qui contribue à la prévention de la transformation des cellules du côlon en cellules cancéreuses [44], Dans notre série, 11,55% de patients étaient alcooliques contre 1,33% des témoins. Nos résultats sont hautement significatifs et concordent avec les données de la méta-analyse de Benoit sur 27 études [45] et des enquêtes épidémiologiques récentes montrant une association positive entre le cancer colorectal et la consommation d’alcool [46, 47].

## Conclusion

Cette étude offre une vision nuancée sur le rôle réellement curatif de l’alimentation sur le risque de cancer colorectal. Nous assistons à une véritable transition épidémiologique avec des changements dans le style de vie, le passage d’une alimentation à base de céréales et de légumineuses à une alimentation riche en viandes, et une consommation de plus en plus fréquente des *«fast food»* et des produits industrialisés. Ces transformations dans notre mode vie font craindre une augmentation de l’incidence des cancers colorectaux dans notre pays, d’où la nécessité d’adopter un régime alimentaire bien codifié. On peut conclure qu’une consommation abondante et variée de fruits et de légumes surtout crus, de fruits secs et oléagineux est essentielle. Rappelons également l’importance du café noir et la nécessité de la consommation bi-hebdomadaire de poissons. La consommation de viandes rouges ou transformés et de pomme de terre cuite ou frites doit rester modérée et il est important de diminuer fortement les aliments de type fast-food, riches en sucres, et graisses saturées, apportant des calories vides. Changer d’alimentation prévient le cancer colorectal, et rééquilibrer la flore intestinale doit faire partie des nouvelles stratégies thérapeutiques pour en stopper la progression.

### Etat des connaissances actuelles sur le sujet

- Le cancer colorectal, par sa fréquence et sa gravité, représente un problème majeur de santé publique au Maroc, l’incidence de ce cancer est en constante croissance comme le prouve l’évolution de l’incidence de ce cancer dans la région du grand Casablanca qui est passée de 7,3 cas/100000 habitants en 2004-2007 à 9,6 cas/1000000 habitants en 2008-2012 pour les deux sexes confondus: il est devenu le premier cancer digestif, surclassant le cancer de l’estomac;
- L’alimentation est considérée comme l’un des plus importants facteurs contribuant au risque de ce cancer, et pourrait rendre compte de 25 à 40% des causes de cancer accessibles à la prévention; elle est le facteur exogène le plus important identifié à ce jour dans l’étiologie du cancer colorectal, on estime que 70% des cancers colorectaux pourraient être prévenus par une intervention nutritionnelle.
- Il est donc essentiel d’étudier la relation entre l’alimentation et la survenue du CCR chez la population marocaine afin de mettre en œuvre une politique de prévention primaire.

### Contribution de notre étude à la connaissance

- Ce travail est réalisé pour la première fois au centre Mohammed VI pour le traitement des cancers l’un des deux plus grands centre pour la prise en charge et le traitement des cancers au Maroc;
- Notre population étudiée est sujette à un déséquilibre alimentaire qui est en faveur du développement ou de la complication de cancer colorectal, d’où la nécessité d’adopter un régime alimentaire bien codifié;
- Nous assistons à une véritable transition épidémiologique avec des changements dans le style de vie, le passage d’une alimentation à base de céréales et de légumineuses à une alimentation riche en viandes, et une consommation de plus en plus fréquente des *«fast food»* et des produits industrialisés; ces transformations dans notre mode vie font craindre une augmentation de l’incidence des cancers colorectaux dans notre pays.

## Conflits d’intérêts

Les auteurs ne déclarent aucun conflit d’intérêts.
